# Proof-of-concept Raman spectroscopy study aimed to differentiate thyroid follicular patterned lesions

**DOI:** 10.1038/s41598-017-14872-1

**Published:** 2017-11-02

**Authors:** Julietta V. Rau, Marco Fosca, Valerio Graziani, Chiara Taffon, Massimiliano Rocchia, Marco Caricato, Paolo Pozzilli, Andrea Onetti Muda, Anna Crescenzi

**Affiliations:** 1 Istituto di Struttura della Materia (ISM-CNR), via del Fosso del Cavaliere 100, 00133 Roma, Italy; 2 Policlinico Universitario Campus Bio-medico, via Álvaro del Portillo 200, 00128 Roma, Italy; 3 Thermo Fisher Scientific, Strada Rivoltana, 20090 Rodano, Milano Italy

## Abstract

Inter-observer variability and cancer over-diagnosis are emerging clinical problems, especially for follicular patterned thyroid lesions. This challenge strongly calls for a new clinical tool to reliably identify neoplastic lesions and to improve the efficiency of differentiation between benign and malignant neoplasms, especially considering the increased diagnosis of small carcinomas and the growing number of thyroid nodules. In this study, we employed a Raman spectroscopy (RS) microscope to investigate frozen thyroid tissues from fourteen patients with thyroid nodules. To generate tissue classification models, a supervised statistical analysis of the Raman spectra was performed. The results obtained demonstrate an accuracy of 78% for RS based diagnosis to discriminate between normal parenchyma and follicular patterned thyroid nodules, and 89% accuracy – for very challenging follicular lesions (carcinoma *versus* adenoma). RS translation into intraoperative diagnosis of frozen sections and in preoperative analysis of biopsies can be very helpful to reduce unnecessary surgery in patients with indeterminate cytological reports.

## Introduction

Patients affected by thyroid nodules are increasing in number, with a concomitant increase of thyroid carcinoma diagnosis and surgery. However, according to the recent literature, in the case of a large number of thyroid nodules, surgical solutions and further treatment can be avoided and therefore represents an over-diagnosis problem. This is particularly true in the case of follicular lesions and of small carcinomas^1^. In the current clinical practice, a significant fraction of patients undergoes total thyroidectomy, often accompanied by neck lymph-node dissection and radiotherapy, without proven benefits. This means that in many cases, thyroid cancer patients receive overtreatment, which subject them to further risks and life-long hormone therapy. This is a serious public health concern, which represents an epidemic scale warning especially in “high-income” countries (up to 90% of cases). It has been recently discussed in the New Engl. J. Med.^2^, by means of an analysis of the available cancer registry data from 12 countries. Therefore, there is an urgent need for a new instrumental tool to reliably assess neoplastic lesions, providing the possibility to combine morphological and biochemical observations.

Follicular patterned thyroid nodules represent a diagnostic problem both at the cytological and histological level. These nodules are known as indeterminate lesions, when observed in cytological samples, and may have low diagnostic inter-observer concordance in the histological setting. The main challenging problem arises when two morphological alterations are present: architectural atypia represented by a microfollicular pattern of growth, and nuclear atypia (the more confusing feature), that raises the possibility of papillary carcinoma, but is too limited or not sufficient to support a diagnosis of malignancy. These features may be present focally or combined, so that the disagreement among observers takes place at a diagnostic level in distinguishing benign (nodular hyperplasia, follicular adenoma) from malignant lesions (follicular variant of papillary carcinoma). Follicular carcinoma, another follicular patterned lesion that embodies the main diagnostic problem in Fine Needle Aspiration (FNA) cytological evaluations, is usually easily recognized based on the histology due the presence of capsular or vascular invasion. However, these tumors also sometimes give rise to diagnostic difficulties due to incomplete evidence of malignant features, so that they are reported as follicular tumors of unknown malignant potential (FT-UMP), without a definitive assessment of the risk to the patient. Thyroid nodules bearing indeterminate cytological findings represent a variable amount (up to 30%) of all FNAs^3^. The indeterminate category is a challenging matter since it is associated with a broad range of malignancy (from 15 to 48%), causing both clinical problems and social burdens^4^. Clinical management of patients with thyroid nodules, undiagnosed following the FNA cytology, is still not resolved and most of these patients are currently referred for diagnostic surgery. Unfortunately, although it is considered the “gold standard”, histological assessment of surgical specimens in the presence of slight nuclear alterations, suffers from great inter-observer variability, due the lack of clear-cut morphological criteria for malignancy^5^. The high inter-observer variability among follicular lesions strongly calls for a new test aimed at diagnostic improvement, in order to avoid unnecessary radioiodine treatment. In the last fifteen years, numerous molecular analyses performed on thyroid nodule tissues have been published, with the scope of reducing the diagnostic variability. Several molecular panels and immunohistochemical tests were developed for this diagnostic purpose^6^. The risk of malignancy, associated with phenotypic changes or different mutational statuses, was obtained by a statistical analysis of frequency that a given alteration is observed in a tumor^7^. Their sensitivity and specificity, however, are variable, and the cost of molecular diagnostics needs to be reduced in order to increase the cost-effectiveness of their use in medical practice.

In this context, Raman spectroscopy (RS) is a particularly suitable label-free technique capable of increasing diagnostic reliability by providing specific biochemical features. Briefly, RS is based on the inelastic scattering of monochromatic light, usually coming from a laser. The laser radiation interacts with a molecular system, resulting in a shift of photon energy, which provides fingerprint information on the structure and the vibrational energy levels. In the last few decades, the number of Raman studies focused on oncology based problems, and more generally on various tissue and cellular pathologies, is growing progressively. Here we cite just a selection of the numerous reviews available in the field^8–14^, of the works of many research groups involved^15–18^, and of the Raman studies dedicated to various organs^19–22^. Various designs of fiber optic Raman probes for real time *in vivo* applications in clinics are discussed in the reviews^23,24^ and in ref.^25,26^. One of the most recent publications in the field describes multifocal Raman micro-spectroscopy allowing “power-sharing” acquisition of Raman spectra from *ad hoc* sampling points^27^.

In this study, we investigated the ability of RS to distinguish between normal parenchyma and follicular patterned thyroid nodules, and between benign (adenoma/hyperplastic nodule) and malignant (follicular carcinoma/follicular variant of papillary carcinoma) patterned lesions. We applied a modern RS microscope which allows both morphological and biochemical characterizations and is applicable to the imaging-mapping of large tissue areas (from μm^2^ to mm^2^). Our aim is to develop a new RS based approach, helpful to reduce the inter-observer variability of the definitive histology, to better plan patient treatment and to prepare the basis for improving pre-operative diagnosis of thyroid pathologies, thus avoiding diagnostic surgery.

## Results

14 patients that received a diagnosis of indeterminate follicular lesion based on FNA at the Endocrinology Unit of the University of Rome “Campus Bio-Medico” (UCBM) were enrolled for this study. Before RS measurements, thyroid tissue sections of 14 patients were histologically identified and diagnosed as healthy or pathological (Haematoxylin/Eosin (H/E) staining of frozen samples) in blind test by three pathologists. The experimental dataset is presented in Table 1, showing the distribution of three sample groups, group N1: healthy thyroid tissues (cases 1–14); group N2: PTC follicular variant (FVPTC) (cases 1–5) and Follicular Carcinoma (FC) (cases 6,7); group N3: Adenoma (Follicular, Macrofollicular, Hyperfunctioning, Oxyphil) and Hyperplastic Colloidal Nodule (cases 8–14).

**Table 1:** Thyroid glands from 14 patients.

Case	Histological diagnosis	No. of healthy tissue maps	No. of pathologic tissue maps
TIR 1	PTC follicular variant	2	2
TIR 2	PTC follicular variant	2	2
TIR 3	PTC follicular variant	2	3
TIR 4	PTC follicular variant	2	2
TIR 5	PTC follicular variant	3	2
TIR 6	Follicular Carcinoma	3	4
TIR 7	Follicular Carcinoma	2	3
TIR 8	Hyperplastic Colloidal Nodule	2	3
TIR 9	Follicular Adenoma	2	3
TIR 10	Follicular Adenoma	2	2
TIR 11	Oxyphil Adenoma	2	2
TIR 12	Oxyphil Adenoma	3	3
TIR 13	Macrofollicular Adenoma	0	4
TIR 14	Hyperfunctioning Adenoma	2	2

Total number of MAPS: 66 Total number of spectra: 28621

### Statistical analysis. Discrimination between healthy and pathologic (carcinoma and adenoma) tissue

The Principal Component Analysis (PCA) was performed, and among the first five PCs (~95%) only PC1 didn’t pass the *t*-test for the difference of the score means for the two typologies of samples (*p*-value of 3.5E-05). Nevertheless, neither singularly nor in combination with the other four PCs, has it been possible to separate the two typologies in a way suitable for diagnostic purposes.

The Linear Discriminant Analysis (LDA) was then applied on the 54 × 5 matrix, obtained considering the scores of the 54 samples for the first PCs chosen. The PCA-LDA model constructed in this way gave as a best result f_1,_ which is a linear combination of PC1, PC2, PC4 and PC5. It gave about 78% of the cases correctly classified.

**Figure 1:**
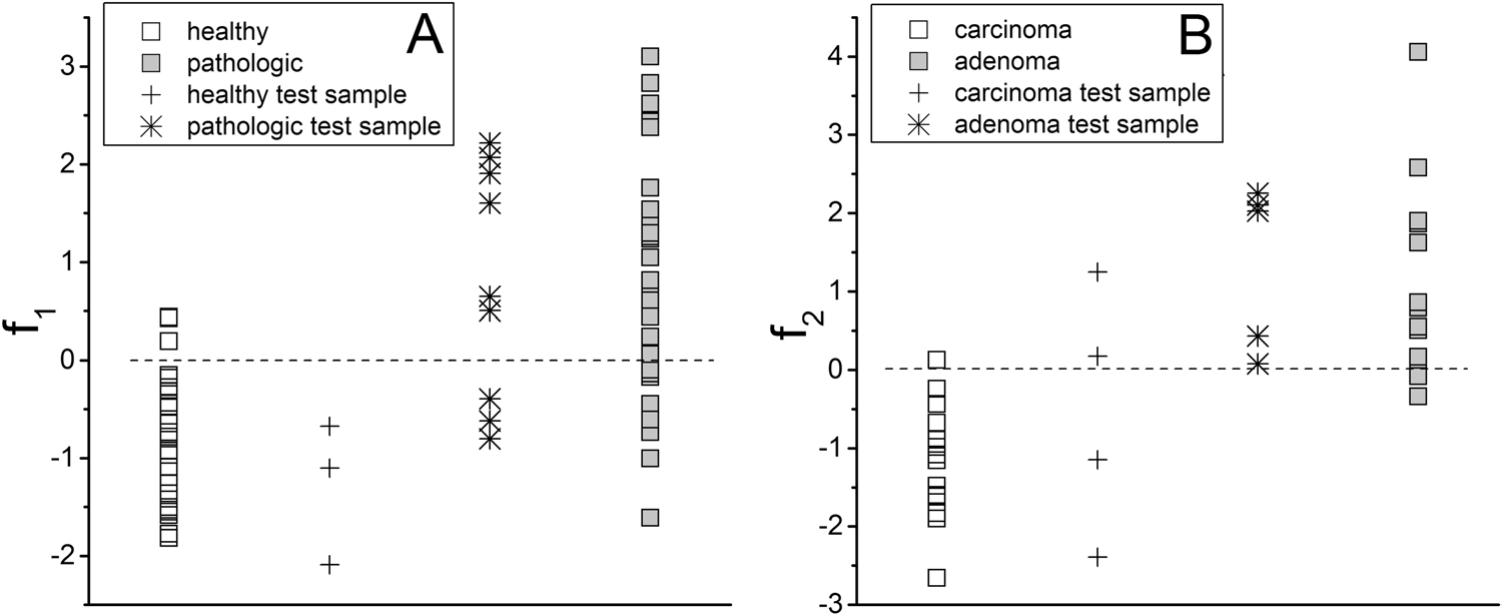
(**A**) projection of healthy and pathologic thyroid tissue samples (including additional independent ones) along f_1_. (**B**) projection of carcinoma and adenoma thyroid tissue samples (including additional independent ones) along f_2_.

A leave-one-out cross-validation method was applied excluding recursively to each spectrum (i.e. average spectrum per map) of the training set and resulted in a 72% of success rate for the correct classification. The scores for f_1_ are shown in Fig. 1(A) (square points). In Supplementary Fig. S1(A), the same data are displayed in a by-thyroid arrangement. As can be seen, healthy tissue samples assume largely negative values of the discriminant function, whereas the majority of the pathological tissues assume positive values. The function f_1_ is less able to classify correctly the pathological samples than the healthy ones. The application of test statistics confirmed the goodness of our results. Having previously verified by Fisher’s test that the variances of the sample scores on f_1_ within the two groups are significantly different (F = 0.278; df_N_ = 25; df_D_ = 27), the two sample *t-*test was applied to the same sample scores, and a value of *t* = −5.983 was obtained for 41.546 degrees of freedom (Welch correction applied). As a result, the two groups shown in Fig. 1(A) are considered significantly different at a very high confidence level («0.001).

A test set (a total of 12 samples obtained from the same patients which provided the samples constituting the training set, cross and star points in Fig. 1(A)) was used as a further tool to test the accuracy of f_1._ Nine samples from 12 were correctly classified. In Table 2 (left part), the results obtained applying the classification model f_1_ and the two validation methods are summarized.

**Table 2:** Results obtained applying classification models f_1_ and f_2_ and two validation methods.

healthy (H) versus pathologic (P) -model f_1_						carcinoma (C) versus adenoma (A) -model f_2_					
Internal validation tool: leave-one-out cross validation			External validation tool: external dataset			Internal validation tool: leave-one-out cross validation			External validation tool: external dataset		
	H	P		H	P		C	A		C	A
H	23	3	H	3	0	C	13	1	C	2	2
P	9	19	P	3	6	A	2	12	A	0	5
	**model f**_**1**_					**model f**_**2**_					
**accuracy**	77.8%					89.3%					
**sensitivity**	88.5%					92.9%					
**specificity**	67.9%					85.7%					

### Discrimination between carcinoma and adenoma

Among the first six PCs (accounting for ~97% of the explained variance) only PC5 didn’t pass the *t*-test (*p* = 1.8E-03). The best result obtained by the cross-validated LDA was the function f_2_ (~89% of correct assessment, ~86% at the cross-validation), combining PC1, PC3, PC4, PC5 and PC6. The scores for f_2_ are shown in Fig. 1(B) (square points). In Supplementary Fig. S1(B), the same data are displayed in a by-thyroid arrangement. Carcinoma samples assume negative values of the discriminant function (one sample misclassified), whereas adenoma samples assume positive values (two samples misclassified). Sample variances were not significantly different (F = 0.374; df_N_ = 13; df_D_ = 13), and the two sample *t*-test gave out a value of *t* = −6.195 for 26 degrees of freedom. As a result, the two groups shown in Fig. 1(B) are significantly different at a very high confidence level («0.001). In this case, the discriminant function f_2_ misclassified two samples of the test set (totally 9 samples obtained from the same patients which provided the samples constituting the training set, cross and star points in Fig. 1(B)). In Table 2 (right part), the results related to the model f_2_ are synthesized.

The results of the statistical analysis described above were obtained by considering an average spectrum per map. To understand if the final results may change, when the statistical analysis is carried out considering a larger number of spectra, we performed an additional study. In order to have the new dataset well equilibrated, the same number of spectra per patient was taken: 200 spectra per each patient (100 healthy and 100 pathologic) and 150 spectra per each patient with pathology. The results obtained are presented in the Supplementary information section (Supplementary Table S1). For the “healthy *versus* pathologic” samples, the results are similar to those obtained considering the average spectra. For “carcinoma *versus* adenoma”, the results are worse than those obtained considering the average spectra. This is likely related to the quality of spectra, which was probably not good enough to enhance the differences between the two pathologies (connected to peak intensities). This effect was less pronounced than in the case of “healthy *versus* pathologic” samples. This will be taken into account in the future experimental studies.

### RS biochemical study

Multiple Raman biochemical maps for the healthy and pathological zones were obtained for each tissue section for 14 patients (see Table 1 for the details).

The following relevant differences of Raman spectral features between carcinoma and healthy tissues were detected:

The 1^st^ group consists of three intense peaks located at Raman shift positions of 1006, 1156 and 1518 cm^−1^. These three peaks are present in the carcinoma Raman patterns (absent in healthy tissues) and represent the spectral fingerprint of carotenoids^28,29^. It should be noted that at 532 nm (the incident radiation wavelength lies in the range of electronic absorption of carotenoids) a significant enhancement of carotenoid bands is observed due to the resonance effect^30^. The 1006 cm^−1^ band is a mixed peak, with contribution from carotenoids and phenylalanine ν_s_(C-C)^28,31^. The less intense 4^th^ carotenoid Raman peak at 956 cm^−1^ was not distinguishable in our spectra, due to its low intensity (only about 10% of the 1156 cm^−1^ band intensity^29^).

The 2^nd^ group of bands, present in the carcinoma spectra, includes peaks located at 1453 and 1667 cm^−1^. We assigned these peaks to proteins, lipids, fatty acids and cholesterol modes, in agreement with the available literature data^8,28,32–34^. Some authors^35^ obtained similar results by collecting Raman spectra of breast cancer epithelial tissue, which are dominated by the same two peaks.

The 3^rd^ group of bands including two peaks at 1223 and 1551 cm^−1^ are present only in the healthy tissue and are attributable to tryptophan, porphyrin, collagen I and nucleic acids modes^28^.

In Supplementary Fig. S3(A), we present the FP range of average Raman spectra (several hundreds or thousands of spectra employed to obtain each average spectrum) obtained for FVPTC (cases 1–5) and FC (cases 6,7). In Supplementary Fig. S3(B), the average spectra corresponding to healthy tissues are shown. The spectrum numbers correspond to the thyroid case numbers given in Table 1.

In order to check for biochemical differences between carcinoma and adenoma presenting follicular patterns, we performed RS investigation of the related thyroid tissue samples. In this case, Adenoma (Follicular, Macrofollicular, Hyperfunctioning, Oxyphil) and Hyperplastic Colloidal Nodule (cases 8–14) were considered. The results obtained are shown in Supplementary Fig. S3(C and D), where the average Raman spectra (several hundreds or thousands of spectra employed for each average) obtained for the related pathological thyroid tissues (Supplementary Fig. S3(C)) and for the healthy tissues of the same thyroid samples (Supplementary Fig. S3(D)) are presented. The spectrum numbers correspond to the thyroid case numbers given in Table 1.

For a better observation of biochemical differences between the 3 groups of tissues, in Supplementary Table S2, an assignment of the thyroid tissue Raman bands registered in our spectra and a comparison with the literature data is given.

The biochemical differences between normal tissue and adenoma can be summarized as follows: adenoma is characterized by the presence of intense signals at 1453 and 1667 cm^−1^ Raman shift positions, whereas healthy tissue is characterized by the absence or weak presence of these bands. The carcinoma spectra show the presence of these two bands as well, although they are less prominent.

A 2^nd^ group of carotenoids peaks (1006, 1156, 1518 cm^−1^) can be detected in some cases, but their intensities are weak compared to those characteristic of carcinoma tissue and are always less intense than the 1^st^ group of bands at 1453 and 1667 cm^−1^.

Finally, the 3^rd^ group of peaks at 1223 and 1551 cm^−1^, respectively assigned to nucleic acids, collagen I and tryptophan, porphyrin modes, are intense and well defined in healthy tissue (Supplementary Fig. S3(D)), while appear strongly depressed, although still detectable, in the pathological ones (Supplementary Fig. S3(C)).

**Figure 2:**
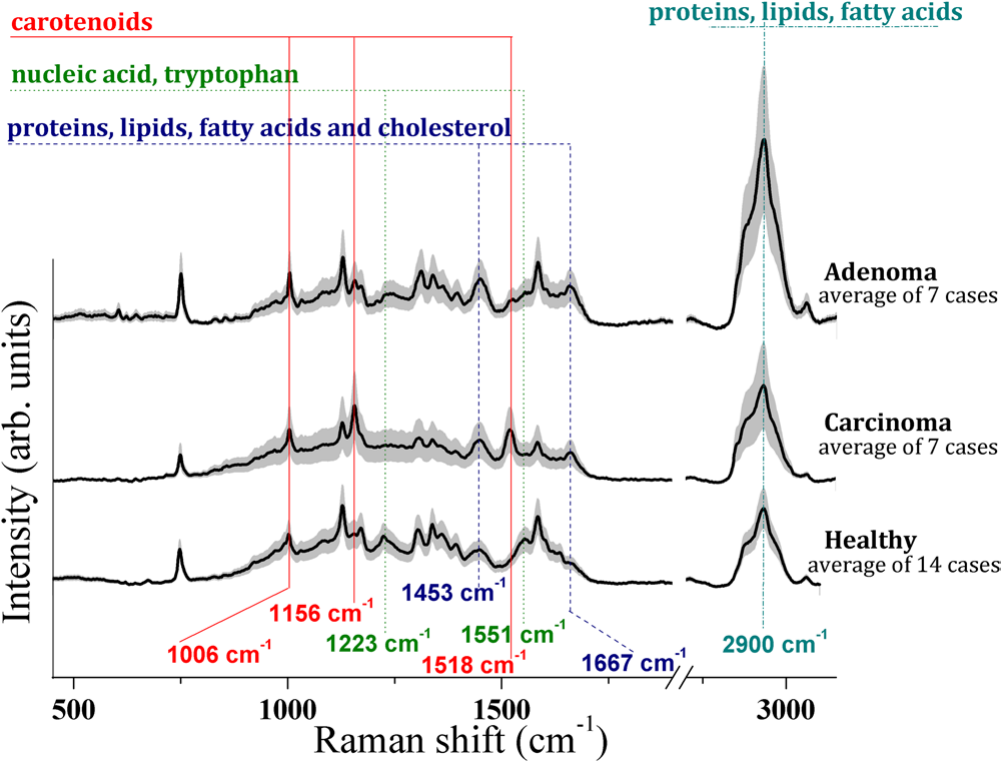
FP and HWN region Raman spectra collected upon healthy and pathologic (carcinoma and adenoma) thyroid tissues. The average spectra ±1 standard deviations from the complete corresponding dataset are shown.

In Fig. 2, the differences between the two main pathologies and healthy tissue can be clearly observed. As can be seen, of the three groups, only carcinoma tissues show the presence of carotenoids bands at 1006, 1156 and 1518 cm^−1^, which are observed in trace quantities in healthy and adenoma tissue; whereas the adenoma tissue’s most important spectral feature is the presence of 1453 and 1667 cm^−1^ bands. The characteristic of healthy tissue is the presence of nucleic acids, collagen I and tryptophan, porphyrin modes at 1223 and 1552 cm^−1^, respectively.

The inter-sample variability can be roughly estimated from Supplementary Fig. S4. The average spectra of thyroid cases (patients) healthy or with the same pathology are rather similar (at least for the presence of the main characteristic peaks) in the corresponding dataset, but nevertheless contain some differences. These differences are mainly related to the bands labeled as a, b, c, d in each of the four panels (A, B, C, D) of Fig. S3. The bands assigned to a- 747 cm^−1^ DNA; b- 1123 cm^−1^ C-N and C-C stretching vibrations of lipids and proteins; c- region between 1300–1400 cm^−1^ tryptophan and normal modes of CH3 and CH2 in lipids, phospholipids, collagen, and nucleic acids; d- 1582 cm^−1^ phenylalanine, are always present in all the tissue types (A–D), but their intensities vary from patient to patient. The mixed Raman peak at 1006 cm^−1^, assignable to carotenoids and phenylalanine, in carcinoma tissue (A) is mainly due to the presence of carotenoids (two other intense carotenoid peaks at 1156 and 1518 cm^−1^ are also present), whereas in other tissue types (B-D) it can be attributed mainly to phenylalanine because the other carotenoid peaks are low in intensity (C) or absent (B, D). The intensity of the 1006 cm^−1^ peak also varies from patient to patient. As for the intra-sample variability, proof of good reproducibility/agreement between the intra-sample spectra, is presented in Supplementary Fig. S4, which shows several examples. As can be seen, for the two different regions of tissue for the case N1, corresponding to the PTC follicular variant pathology, the 2 average spectra shown are almost identical, although the standard deviations are not negligible. The same can be testified for the 3 average spectra representing three different regions of the N12 case (adenoma) and two different healthy tissue regions of the N2 case. A more detailed study is planned to further investigate the inter- and intra-sample variability issues.

**Figure 3:**
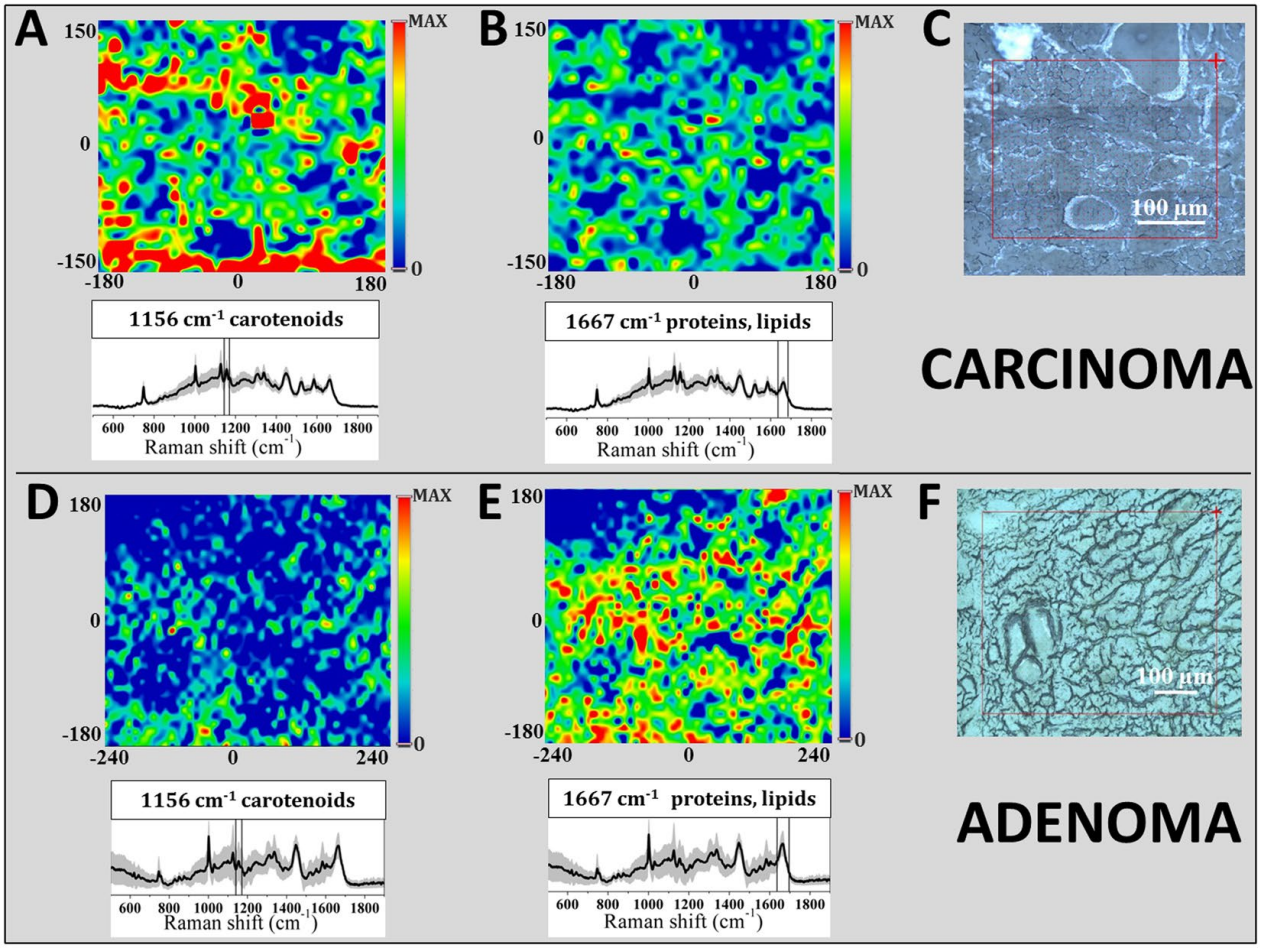
Raman chemigram maps in false colors for thyroid tissues. In the upper part, carcinoma tissue is presented: (**A**) chemigram is obtained considering the area of the 1156 cm^−1^ band, (**B**) chemigram with 1667 cm^−1^ band reference, (**C**) dark field optical image). In the lower part, adenoma tissue is shown: (**D**) chemigram with 1156 cm^−1^ band reference, (**E**) chemigram with 1667 cm^−1^ band reference, F- dark field optical image). The chemigram scale bars are expressed in μm. The average Raman spectra ± 1 standard deviations are presented below each map. The red squares on the dark field optical images correspond to the investigated tissue areas. The step size for both images is 10 µm (about 1500 points).

The above described biochemical differences between adenoma and carcinoma can be easily observed in Fig. 3, where Raman chemigrams with respect to the two arbitrarily chosen reference bands are shown: one is the 1156 cm^−1^ carotenoid peak and the other is the 1667 cm^−1^ proteins, lipids, fatty acids and cholesterol Raman intensity. The false color scale bars, present on the right of each chemigram, show the correspondence between colors and intensity. For carcinoma tissue, chemigram A is obtained considering the area of the 1156 cm^−1^ band (the red color represents the highest peak intensity, while the blue shows the absence of the corresponding peak). In an analogous way, chemigram B is obtained considering the area of the 1667 cm^−1^ band (red corresponds to the maximum intensity and blue to the minimum). For adenoma tissue, chemigrams are prepared in the same way: D- with 1156 cm^−1^ band reference, E- with 1667 cm^−1^ band reference).

In the dark field image from carcinoma (C) (see Fig. 3) the follicular architecture of the neoplastic tissue is evident and is characterized by the presence of carotenoids (A), represented in red to yellow. In the image B, the protein and lipid constituents are evenly distributed throughout the tissue with the exception of vessels lumen clearly distinguishable in blue. In the dark field image from the Hurthle cell follicular adenoma (F), the carotenoids are not detectable (D), while proteins and lipids are present in abundance within the tissue (E).

**Figure 4:**
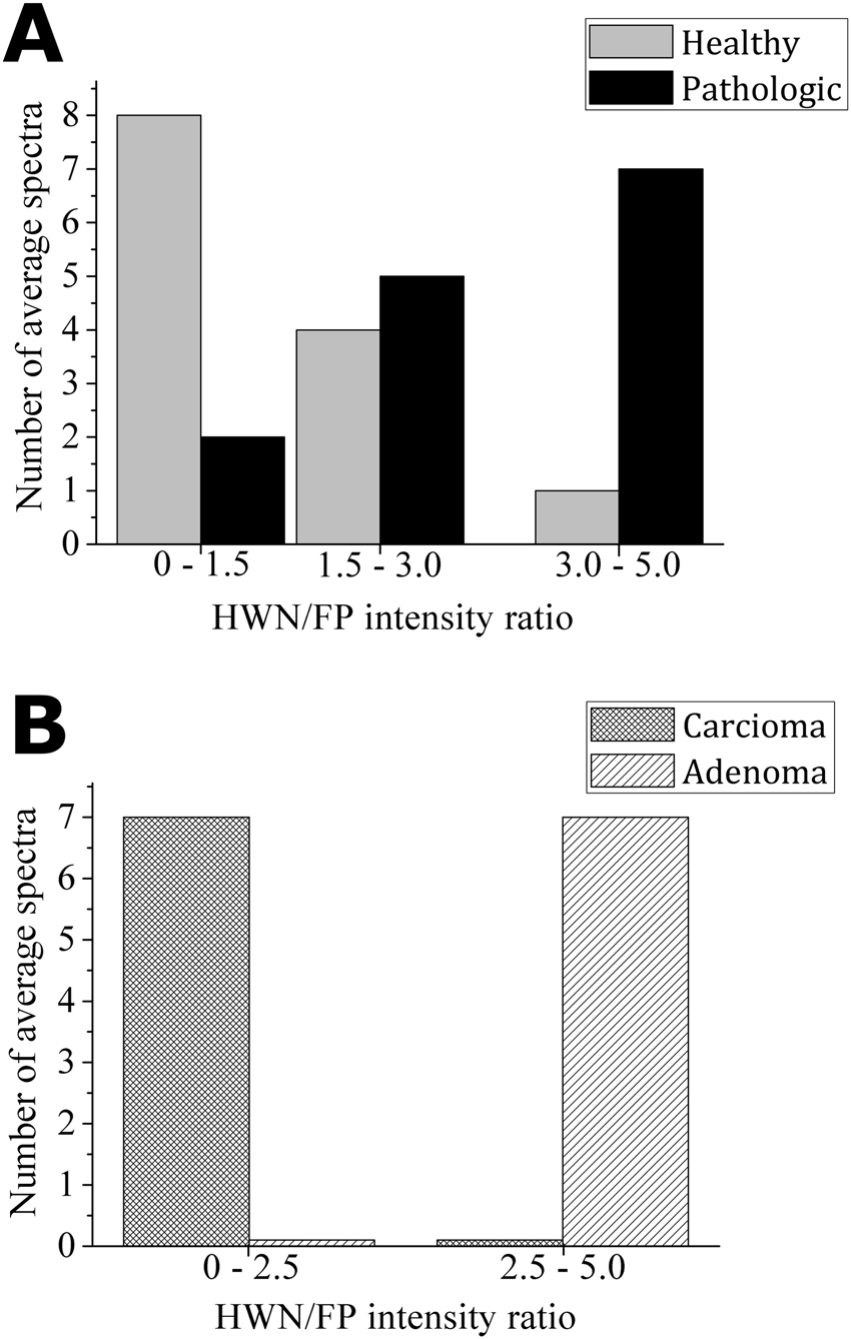
(**A**) HWN/FP intensity ratio distribution in healthy *versus* pathologic tissues and (**B**) HWN/FP intensity ratio distribution in carcinoma *versus* adenoma.

In addition, the HWN region of Raman spectra was carefully analyzed (see Fig. 2). The presence of a broad band centered at 2900 cm^−1^, generally assigned to proteins, lipids and fatty acids vibrational modes, can be observed in all the spectra^28^. It should be noted that on sorting the pathologic tissue spectra according to their HWN/FP ratio in decreasing order, they self-separate into two groups as the adenoma tissue is characterized by a higher HWN/FP ratio. In order to better appreciate the different HWN/FP ratio values, characteristic for healthy, adenoma and carcinoma tissue, the experimental data obtained are presented in the form of histograms in Fig. 4(A and B). In Fig. 4(A), the HWN/FP intensity ratio distribution in healthy *versus* pathologic tissue is plotted. It can be seen that the higher HWN/FP ratio is more characteristic for pathologic tissues, while the healthy tissues are characterized by lower values. In Fig. 4(B), the histogram for carcinoma *versus* adenoma is plotted, and the latter pathology is characterized by higher values.

## Discussion

In this work, we analyzed the most evident biochemical differences between the 3 groups of thyroid tissues. Based on the experimental results obtained, it can be confidently stated that only carcinoma tissues are characterized by the significant presence of carotenoids, which are absent or present in trace quantities in healthy and adenoma tissues. This result is in agreement with our previous study, where the presence of a significant amount of carotenoids in thyroid neoplasms has been reported^36^.

For the adenoma tissue, the most important spectral feature is the presence of the 1453 and 1667 cm^−1^ bands, assigned to proteins, lipids, fatty acids and cholesterol modes. The Raman spectra of healthy thyroid tissues show features due to tryptophan, porphyrin, collagen I and the (PO_2_^−^) group of nucleic acid bands.

In the HWN range of spectra, where Raman shifts of proteins, lipids and fatty acids are registered, the most interesting experimental evidence is the fact that pathologic thyroid tissues spectra self-separate into two groups, because adenoma tissues are characterized by a higher HWN/FP ratio with respect to the carcinoma tissues, whereas healthy tissues are characterized by a lower corresponding ratio, in agreement with our previous work^36^.

The Raman spectroscopic approach, which provides biochemical information, exceeds the diagnostic analysis based on morphological features, due to the challenging context, particularly for follicular patterned thyroid nodules. Differentiated thyroid tumors with follicular patterns represent a diagnostic problem both at the first assessment of patients with thyroid nodules, relying on cytological evaluation, and at the definitive histology on the surgical sample. Cytological features of malignant follicular neoplasms, namely FC and FVPTC share morphological aspects with benign nodular lesions and in many cases require diagnostic surgery. Histological identification of FC can be easy, when capsular or vascular invasions are evident, whereas this assessment may be challenging when the diagnostic features are incomplete or subtle, and a follicular adenoma (FA) cannot be excluded. In the same way, FVPTC can be easily diagnosed when nuclear features of PTC are classic and diffusely distributed throughout the tumor, while distinguishing FVPTC from a benign adenoma or a hyperplastic nodule can be extremely difficult if nuclear alterations are not well developed or are only focally expressed. Currently, there is a considerable inter- and intra-observer variability in the diagnosis of FVPTC, likely, due to the lack of agreement on the minimal criteria needed to diagnose FVPTC, even among the experts^37^. Various compounds, such as galectin3 and HBME1, were observed, as they are expressed in differentiated carcinomas, and are proposed as diagnostic and prognostic markers by the immunohistochemistry^38^. Their presence, however, is not restricted to malignancy as they are also identified in some benign conditions. Moreover, the extensive assessment of the molecular landscape of differentiated thyroid tumors has not been translated into the diagnostic routine of follicular patterned lesions, due to a low rate of sensitivity and specificity for this kind of setting^39^.

RS is a very promising tool for biological tissue studies, and it has recently been employed also for various clinically driven investigations as a potential diagnostic tool^10–14,40^. RS investigations of thyroid, described in the literature, regard cancerous and non-cancerous cell lines^41,42^, normal thyroid tissues^31,43^ and some thyroid tissue pathologies, such as thyroid cancers and nodular goiters^31,43^, goiters, follicular adenoma, papillary and follicular carcinoma^44^ Our previous study^36^, for the first time, reported the Raman fingerprint of PTC, characterized by significant presence of carotenoids with respect to the healthy tissue, obtained on unlabeled frozen tissue sections, better preserving in this way the molecular content of the cells. Performing coupled histopathologic and Raman biochemical observations, we demonstrated that RS reliably identifies PTC with a high specificity and sensitivity. In the present work, in the context of follicular patterned nodules, we identified specific spectral profiles for non-neoplastic nodules, benign neoplasms and carcinomas. The spectra obtained for hyperplastic microfollicular lesions and follicular adenoma were significantly different from those obtained for healthy tissue, and both are clearly distinguishable from the spectra obtained for FC and FVPTC.

Our combined histological and Raman spectroscopy approach allows a more accurate identification of lesions with malignant potential. An important consequence of the improved combined diagnosis can be the avoidance of unnecessary radioiodine treatment and close clinical surveillance, accompanied by a significant reduction of health costs. Finally, we suggest the use of Raman microscopy for intra-operative diagnosis of thyroid nodules. To date, intra-operative frozen sections are not recommended by major international guidelines in follicular lesions due the low sensitivity and specificity of this method in discriminating between benign and malignant nodules^45,46^. In this work, we demonstrated that RS analysis of untreated and unstained frozen thyroid sections can achieve diagnostic accuracy, sensitivity and specificity that are compatible with clinical use, and it can be reliably translated into intra-operative diagnostics to support correct decision-making in surgical strategy (i.e. lobectomy *versus* total thyroidectomy). This application is in agreement with the recent indication to reduce extensive surgery, strongly suggested by the international guidelines^47^.

In conclusion, this proof-of-concept Raman study is an important step toward the increase of the reliability of the diagnosis of follicular thyroid lesions, by coupled histopathologic and RS investigations. The results show about 78% of accuracy for the RS based diagnosis in distinguishing between healthy and neoplastic tissue, while for very challenging follicular lesions (carcinoma *versus* adenoma), the obtained results exhibit about 89% of accuracy. This work demonstrates the great potential of RS biochemical fingerprints to contribute to clinical decision-making. The RS translation into the pre-operative bioptic sampling process in order to reduce unnecessary surgery in patients with indeterminate cytological reports can be very helpful. Finally, the evaluation of Raman spectra may allow the identification of molecular species involved in thyroid tumorigenesis and progression.

## Materials and Methods

### Ethics statement

This prospective monocentric study was approved by the Ethical Committee of the UCBM (prot. 33.15 TS ComEt CBM). The informed undersigned consent was collected from patients before surgery. Enrolled patients are known to the pathologist and were recorded in a codified file with an anonymous ID code, which was also registered in the software database of the Pathology Unit of the UCBM, containing personal identifiable information. All experiments were performed in full accordance with the principle of Good Clinical Practice and the ethical principles, contained in the current version of the Declaration of Helsinki.

### Thyroid tissues

14 patients that received a diagnosis of indeterminate follicular lesion based on FNA at the Endocrinology Unit of the UCBM and were candidate for surgery were enrolled for this study. Indeterminate cytology was referred to indeterminate cytological atypia as well as to architectural follicular atypia or similar subclasses in comparable cytological classification systems^46,48^. These patients underwent total thyroidectomy at the Surgery Unit of the UCBM. At the time of surgery, the specimens removed were immediately submitted unfixed to the Pathology Unit of the UCBM in an appropriately labeled container. After completion of the initial examination of the specimen by the pathologists, resection margins were marked with black ink. Pathological sampling was carried out in agreement with international guidelines for handling surgical specimens^49^. A tissue slice of about 1 × 1 × 0.3 cm^3^ was then obtained, normally including both healthy and neoplastic areas, avoiding surgical margins. It was frozen on a metallic cold-plate inside the cryostat. A 5 µm cryostatic section was cut and stained with H/E, in order to confirm the presence of healthy and neoplastic tissue zones, as well as the transition area between them. Additional consecutive sections for RS studies were cut at 20 and 30 µm of thickness, collected on separate slides and stored unstained at −20 °C until the Raman evaluation. The surgical samples were subsequently fixed in buffered formalin and embedded in paraffin for permanent sectioning. Diagnosis, grading and staging were performed, in agreement with the 7th edition of TNM (Tumor, Node, and Metastasis)^50^. To overcame the problem of inter-observer variability in the setting of follicular thyroid nodules, in our study, all cases were diagnosed in blind by three pathologists (AC, AOM, CT) with extensive experience in thyroid pathology. In this activity, the pathologists were supported by the immunohistochemical stains with antibodies to Galectin 3, CK 19, HBME1 and CD56. At least two immunomarker alterations were observed in cases reported as carcinoma. Moreover, in difficult cases of the encapsulated follicular variant of papillary carcinomas, the diagnostic evaluation was standardized using the nuclear score, as recently proposed^51^. As a result of this multifactorial approach all cases enrolled for this study had complete concordance among the pathologists. To confidentially measure tissue regions that were associated with healthy and pathologic changes, we compared, for each sample, the section stained with H/E for light field microscopic evaluation and the thick unstained section. We encircled  with a permanent ink pen the pathologic area in the stained slide under light field microscopy; then we identified the same area in the unstained section by confronting the slides. In the unstained slide, the pathologic area was evidenced by diamond tip indelible pen (Thermo Scientific™ Diamond Point Marker). Areas for Raman spectra acquisition were selected within and out of the pathologic regions; these areas were also verified in dark field microscopy before Raman scanning.

### Raman spectroscopic measurements

No pre-treatment was performed on tissue samples before spectroscopic Raman examination. Raman spectra were recorded using a Thermo Fisher Scientific DXR Raman microscope at the following conditions: 532 nm laser source; 200–3400 cm^−1^ full range grating; 10x and 50x objectives; 25 μm confocal pinhole, 5 (Full Width Half Maximum) cm^−1^ spectral resolution. As a first step, the collection of a number of mosaic images at low magnification (10x) has been carried out, providing the generic overview information on tissue morphology and allowing one to individuate and evaluate regions of interest. After that, the area of interest was investigated, collecting spectra at high magnification (50x). All the spectra/maps presented in this work were taken with 50x objective. A 5^th^ order polynomial correction via the modified polyfit was used to compensate tissue fluorescence^52^. A laser power of 8 mW, measured at the sample, has been applied, as the best compromise between the signal quality and the undesired tissue burning. The exposure time was 0.8 s, as a suitable compromise to achieve a good spectra quality and to shorten the overall acquisition time. At least 50 exposures were averaged for better Signal-to-Noise Ratio (SNR). Raw spectra are characterized by a SNR ranging from 27 to 40. Average spectra, representative of the entire region of tissue, being the results of a post-elaboration averaging of hundreds or thousands of spectra, are characterized by a SNR ranging from 50 to 63. Laser spot size was about 700 nm (50x objective). Raman maps of tissue areas from approximately 100 × 100 μm^2^ up to 1 × 1 mm^2^, collecting from several hundreds up to several thousands of spectra per map, were obtained. The time scan for 1 map at 10 µm step size, containing about 1500 points, was about 12 h, while for 1 map at 50 µm step size, containing about 200 points, was approximately 1 h 30 min. To assess intra-sample variability, multiple measurements were carried out at different regions on the same tissue.

### Statistical analysis

The statistical analysis was performed considering the same number of Raman maps for each patient (two healthy and two pathologic) and for each tissue type. The average spectra obtained from each map were considered in the ranges of 653–1723 cm^−1^ (fingerprint (FP)) and of 2828–3023 cm^−1^ (High Wave Number (HWN)).

At first, PCA was carried out in order to reduce the initial dimensionality of the dataset and to verify whether, in a subset of dimensions, the internal variability of the spectra can reveal some differences among tissue types that can be considered diagnostic.

LDA was applied on the same components observed in PCA. Moreover, the implementation of the algorithm on principal components (PCs) let us to optimize the process of classification on the basis of a reduced number of input variables. The LDA was tested using the leave-one-out cross validation (internal validation tool) and an external test set.

### Discrimination between healthy and pathologic (carcinoma and adenoma) tissue

The PCA was performed on a matrix 54 × 1314 of the 54 average spectra (54 = 13 × 2 + 14 × 2), corresponding to 54 Raman maps obtained for tissue samples, in the FP and HWN regions. Two pre-treatment procedures were applied before PCA: normalization of the average spectra for the area under the curve, used to correct the differences in the global intensity and make them more comparable, and auto-scaling on the columns.

### Discrimination between carcinoma and adenoma

The PCA was performed on a matrix 28 × 1314 of the 28 average spectra (28 = 7 × 2 + 7 × 2), corresponding to 28 Raman maps obtained for tissue samples in the FP and HWN regions. The same pre-treatment procedures of the previous paragraph were applied here.

### Data Availability

The datasets generated and/or analyzed during the current study are available from the corresponding author on reasonable request.

## Electronic supplementary material

Supplementary Information
