# Supplementary material for: Proof-of-concept Raman spectroscopy study aimed to differentiate thyroid follicular patterned lesions
Source: Sci Rep. 2017 Nov 2;7:14970. doi: 10.1038/s41598-017-14872-1 (PMC5668290; doi:10.1038/s41598-017-14872-1)

Proof-of-concept Raman spectroscopy study aimed to differentiate thyroid follicular patterned lesions

Julietta V. Rau[a]*, Marco Fosca[a], Valerio Graziani[a], Chiara Taffon[b], Massimiliano Rocchia[c], Marco Caricato[b], Paolo Pozzilli[b], Andrea Onetti Muda[b], Anna Crescenzi[b]

[a]*Istituto di Struttura della Materia (ISM-CNR), via del Fosso del Cavaliere 100, 00133 Roma, Italy*
[b]*Policlinico Universitario Campus Bio-medico, via Álvaro del Portillo 200, 00128 Roma, Italy*
[c]*Thermo Fisher Scientific, Strada Rivoltana, 20090 Rodano, Milano, Italy*

***Corresponding author:** Julietta V. Rau, Istituto di Struttura della Materia (ISM-CNR), via del Fosso del Cavaliere 100, 00133 Roma, Italy. Phone: + 39-06-4993-4086; Fax: + 39-06-4993-4153
Email: giulietta.rau@ism.cnr.it

Supplementary Fig. S1. **A-** projection of healthy and pathologic thyroid tissue samples (including additional independent ones) along f_1 by progressive thyroid number. **B-** projection of carcinoma and adenoma thyroid tissue samples (including additional independent ones) along f_2 by progressive thyroid number.

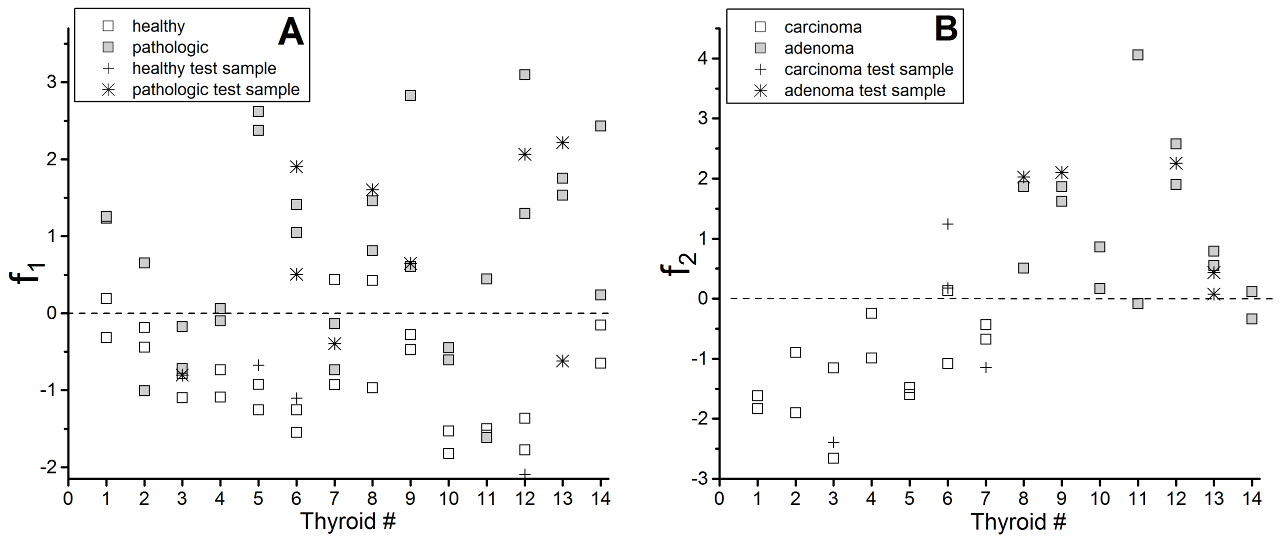

Supplementary results: statistical analysis of the thyroid follicular patterned lesions - a different approach

In addition to the statistical results presented in the paper, a new statistical analysis was performed on a larger training dataset in order to employ a higher number of spectra. The main sources of this variability are the spectral noise (no longer reduced by the averaging) and the presence of spectra collected in non-optimal focus condition, due to the non-homogeneous topography of samples (resulting in non well defined peaks and random distortions induced by the algorithm of polynomial correction).

For both the "healthy *versus* pathologic" and the "carcinoma *versus* adenoma" cases, a criterion of equality has been applied to obtain a well-equilibrated new dataset. The same number of spectra per patient, equally distributed between a couple of maps for each case for each patient, was taken: in the first case, 200 spectra per each patient (100 healthy and 100 pathologic); in the second case, 100 spectra per patient with pathology. All these spectra were obtained by a systematic grid sampling of the hyperspectral images.

The pre-treatment of the data and statistical procedures were the same as in the main text. The FP and HWN spectral ranges considered were the same as defined in the main text. The two build PCA-LDA models were tested both by the leave-one-patient-out cross validation and a test set, constituted by a sub-set of the patient data from which also the training set was derived.

Discrimination between healthy and pathologic tissue. In this case, the first principal components assume lower values with respect to the study carried out on the average spectra, as a consequence of the increased number of sources of variability present in the dataset (for comparison, the ~95% of the explained variance is reached at the 11th PC). From the analysis of the first PCs accounting for the 90%, PC1, PC2 and PC3 didn't pass the *t*-test for the difference of the score means for the two sample typologies (*p*-values «0.001). In particular, for PC1, the *p*-value is very low (*p*«3E-83). The LDA was then applied on the score samples, and the best result (f_{1bis}) was obtained for a linear combination of PC1, PC2, PC3 and PC5, giving about 72% of the cases correctly classified. The validation by means of the test set is shown in Supplementary Table S1 (left part). These results are in good accordance with the ones obtained for the corresponding model f_1. A comparison of the loadings of the two models (f_1 and f_{1bis}) is shown in Fig. S2(A), where a common trend and the presence of peaks identified in the biochemical study are well recognizable.

Discrimination between carcinoma and adenoma tissue. Also in this case, the first principal components assume lower values with respect to the study carried out on the average spectra, and the ~95% of the explained variance is reached at the 10th PC. All of the first PCs accounting for the 90% didn't pass the *t*-test for the difference of the score means for the two typologies of samples, but only PC1, PC3, and PC5 reach a value as low as the one corresponding to a recognizable separation in a graphical representation of the scores (respectively, *p*-values «3E-29, 3E-33, 5E-19). The LDA was then applied on the score samples and the best result (f_{2bis}) was obtained for a linear combination of the first five PCs plus the seventh, giving about 61% of the cases correctly classified. The validation by means of the test set is shown in Supplementary Table S1 (right part). Even if these results are worse than the ones obtained applying the corresponding model f_2, f_{2bis} still shows an equilibrium between sensitivity and specificity. In Fig. S2(B), a comparison of the loadings for f_2 and f_{2bis} is shown. Even if with some differences at certain wavenumbers (777, 1006, 1337, 1486, 1602 cm^{-1}), both the trends have common features and the peaks identified in the biochemical study are well recognizable.

Supplementary Table S1. Results obtained applying classification models f_{1bis} and f_{2bis}.

healthy (H) *vs* pathologic (P) - model f_{1bis}						carcinoma (C) *vs* adenoma (A) - model f_{2bis}					
Internal validation tool: leave-one-patient-out cross validation			External validation tool: test set			Internal validation tool: leave-one-patient-out cross validation			External validation tool: test set		
	H	P		H	P		C	A		C	A
H	982	316	H	450	42	C	401	299	C	276	80
P	449	953	P	313	667	A	241	459	A	132	492
**model f_{1bis}**						**model f_{2bis}**					
**accuracy**	71.7%		75.8%			**accuracy**	61.4%		78.4%		
**sensitivity**	75.7%		91.5%			**sensitivity**	57.3%		77.5%		
**specificity**	68.0%		68.0%			**specificity**	65.6%		78.8%		

The values of the estimation parameters of both f_{1bis} and f_{2bis} are reasonably lower in the case of the cross-validation with respect to external validation, due to the fact that the latter dataset was extracted from the same patients used for the training set, resulting this in an overoptimistic evaluation. In the "healthy *versus* pathologic" case, the discrepancy between the sensitivity and the specificity from the external validation and the considerable difference of the sensitivity values for the two validation methods (Table S1, left part) suggests that the best model f_{1bis} obtained applying the PCA-LDA methods overfits the data. The much more coherent accuracy, sensitivity and specificity values of the cross validation appear more realistic. In the "carcinoma *versus* adenoma"

case, for each validation method, sensitivity and specificity show similar values, but the discrepancy between the results of the methods (Table S1, right part) evidences that the model f_{2bis} overfits the data.

Fig. S2. A- comparison between the loadings of f_1(average spectra) and of f_{1bis} (downsampled image spectra); B – comparison between the loadings of f_2(average spectra) and of f_{2bis} (downsampled image spectra).

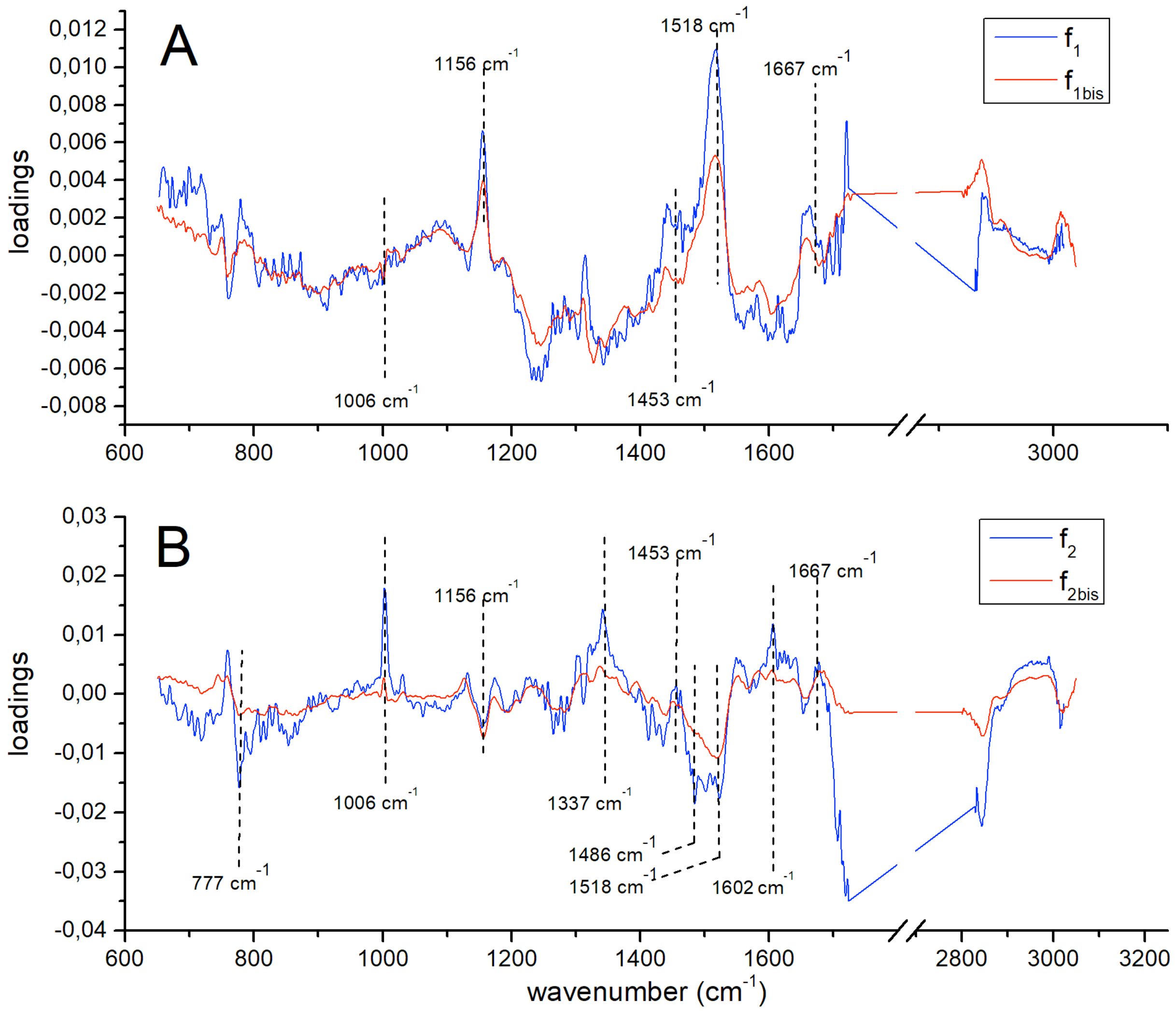

The obtained results regarding the quality of the developed models considering average spectra and large number of spectra from hyperspectral images suggest that, in the latter case, other approaches of statistical analysis should be employed to reach biomedical-level accuracy.

In fact, in homogeneous cell samples, such as those employed in our study, the major part of variability of the hyperspectral images collected using the Raman microscope is related to the variety of focus conditions. All this resulted in a negative effect on the PCA-based statistical approach. On the contrary, the average spectra are not effected by the same issue and more stable and accurate PCA-LDA models can be obtained.

Point-by-point focus collection is permitted by the present-day technology, but still requires long collecting times and, consequently, the availability of fresh biological samples. All these issues will be taken into account in the setting of future approaches to these studies.

Supplementary Fig. S3. FP spectral region: **A**- sequence of average Raman spectra collected upon PTC follicular variant and FC thyroid tissues (cases 1-7); **B** - sequence of average Raman spectra collected upon corresponding healthy tissues. **C**- sequence of average Raman spectra collected upon Adenoma (Follicular, Macrofollicular, Hyperfunctioning, Oxyphil) and Hyperplastic Colloidal Nodule (cases 8-14), **D**- sequence of average Raman spectra collected upon corresponding healthy tissues. Spectrum numbers correspond to the thyroid case/patient numbers given in Table 1.

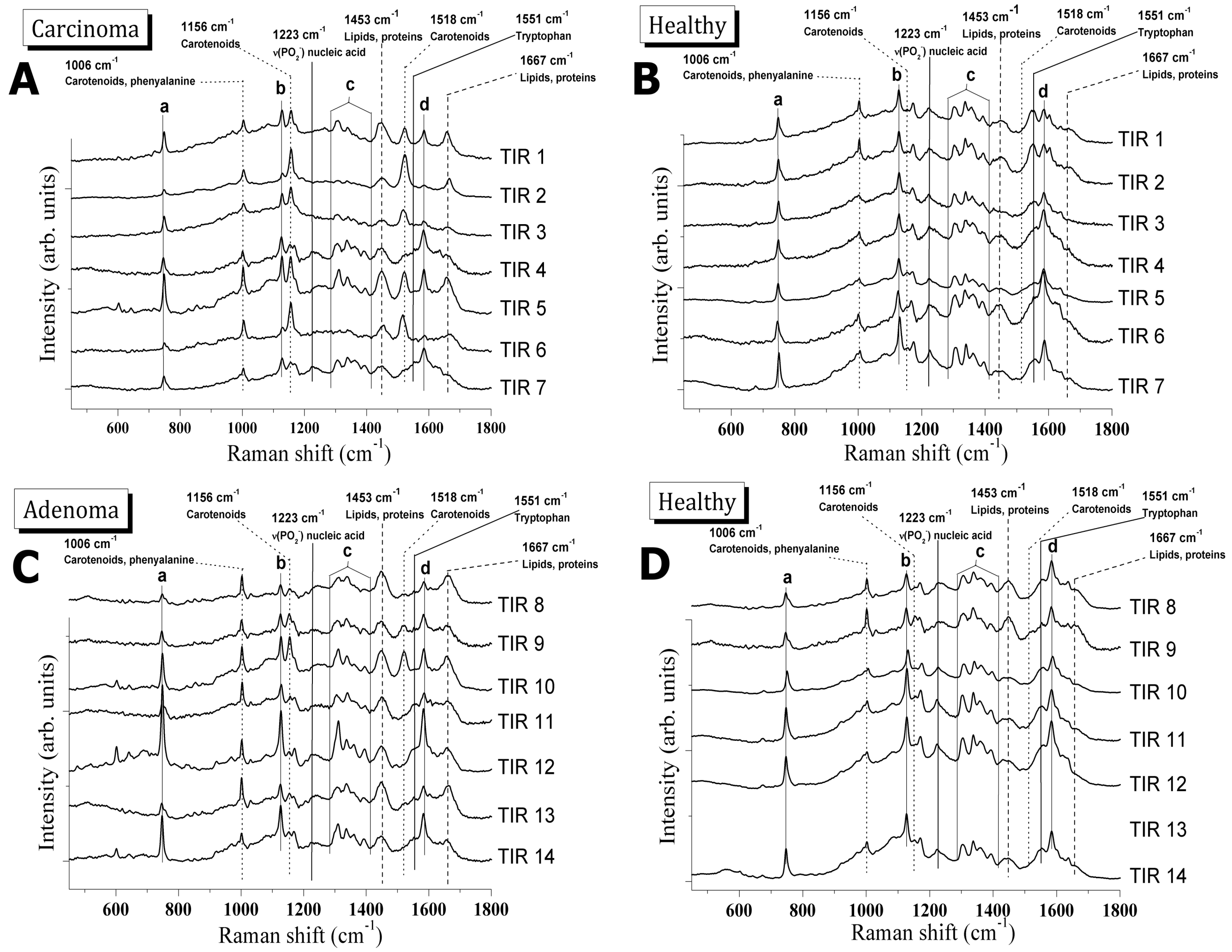

Supplementary Table S2. Peak positions and assignments of Raman bands observed in healthy and pathologic thyroid tissues.

**Healthy tissue peak position (cm^{-1})**	Follicular PTC/ Follicular Carcinoma tissue peak position (cm^{-1})	Follicular Pattern Adenoma peak position (cm^{-1})	Band attribution	**Reference**
		603	Unassigned band	
		640	C-S stretching and C-C twisting of proteins; Tyrosine	(28)
673		673	Tryptophan (ring breathing)	(28)
	717		Membrane phospholipids head (C-N); Adenine; Lipids ($CN^{+}(CH_3)_3$)	(28)
748	748	748	DNA; Tryptophan	(28)
		825	O-P-O stretch DNA	(28)
		851	Proline&hydroxyproline (side chain vibration); Tyrosine (ring breathing and Fermi doublet); Glycogen	(28, 44)
919	919	919	Proline; Hydroxyproline; Glycogen; Lactic acid	(28)
	957		Carotenoids; Phosphates $\nu_s(PO_4^{3-})$; Cholesterol; Quinoid ring in-plane deformation	(28, 44)
971	971	971	ν(C-C) wagging	(28)
994		994	C-O ribose; C-C	(28)
1006	1006	1006	Phenylalanine ν_s(C-C); Carotenoids	(28, 31)
1031	1031	1031	Phenylalanine (δ(C-H) and C-H in plane bending); Protein (C-N stretching); Carbohydrate residues of collagen	(28, 44)
1080	1080	1080	Amide II; Typical phospholipids; Phosphate vibrations; Collagen; Tryptophan	(28)
1086	1089	1089	ν(C-C) *gauche*;$\nu_1(CO_3^{2-})$; $\nu_3(PO_4^{3-})$; $\nu(PO^{2-})$; ν(C-C) skeletal of acyl backbone in lipid (*gauche* conform.)	(28, 44)
1128	1128	1128	Proteins (C-N stretching); Carbohydrates (C-O stretching); Ceramides; Acyl backbone in lipid (trans conform., ν(C-C))	(13, 31, 44)
	1156	1156	Carotenoids	(28)
1172	1172	1172	δ(C-H); Tyrosine	(28)
1208		1208	$\nu(C–C_6H_5)$; Tryptophan; Phenylalanine (protein assignment); A, T (ring breathing modes of the DNA/RNA bases); Amide III (protein); Tryptophan	(28, 44)

			and phenylalanine ν(C C6H5) mode	
1220-1300		1220-1300	Amide III (β sheet structure)	(28)
1223			ν(PO_2^-), nucleic acids; Cellular nucleic acids; A concerted ring mode; Proteins, including collagen I	(28)
1234		1234	A concerted ring mode	(28)
1239		1239	Amide III	(28, 44)
		1255	Lipids; A, T (ring breathing modes of the DNA/RNA bases); Amide III (protein)	(28)
	1264		Lipids	(28)
1307	1307	1310	Lipid and collagen (twisting, bending, wagging)	(28)
1337	1337	1337	C-H deformation (protein); Amide III; Glycine and proline side chain (CH_2 wagging vibrations); Adenine and guanine (ring breathing modes)	(28)
1360	1360	1360	Tryptophan	(28)
1393	1393	1393	CH rocking	(28)
1424	1424	1424	Lipid (CH_2 scissoring); Deoxyribose (B, Z-marker);	(28)
1442	1442	1442	CH, CH_2 and CH_3 deformation; Cholesterol; Triglycerides (fatty acids); Lipids (CH_2 scissoring and CH_3 bending); Collagen	(28)
1445	1453	1448	Collagen (δ(CH_2), δ(CH_3) and CH_2CH_3 bending); Phospholipids (δ(CH_2), δ(CH_3) and CH_2CH_3 bending); Methylene (bending); ν(C-H); δ(CH_2) of lipids/proteins; Methyl groups bending; Proteins (δ(CH) and δ(CH_2)); Protein bands; C-H bending mode of structural proteins; Structural proteins of tumors 1430÷1470 cm^{-1} (CH_2/CH_3) bending and (CH_2/CH_3) scissoring of membrane lipids, fatty acids, cholesterol and cholesterol ester	(28, 32-35)
	1518	1518	(-C=C-) carotenoids	(28)
1551			Tryptophan ν(C=C); Porphyrin ν(C=C)	(28)
1557			Tryptophan; Porphyrin ν(C=C); Amide II (ν(CN) and δ(NH)); COO− tyrosine, amide II);	(28)
1584	1584	1584	Phenylalanine δ(C-C); (C-C) olefinic stretching; Hydroxyproline; Acetoacetate;Riboflavin; Lipids	(28, 31, 44)
1602	1602	1602	Phenylalanine δ(C-C)	(28)
1638		1638	Water (intermolecular bending and very weak and broad ν2)	(28)
1660			ν(C-C) cis; (C-C) groups in unsaturated	(28)

			fatty acids; Fatty acids; Lipids; Ceramide backbone; Amide I	
1662			Nucleic acid modes; Nucleic acid modes indicating the nucleic acid content in tissues	(28)
	1667	1667	Protein band; C=C stretching band; α-Helical structure of amide I; Structural protein modes; Carbonyl stretch (C=O); ν(C=C) of lipids; ν(C=C) of membrane, fatty acids, cholesterol and cholesterol ester	(28, 32, 34-35)
	2852		$\nu_s(CH_2)$; Lipids; Fatty acids	(28)
2879		2879	Lipids and proteins (CH_2 and CH)	(28)
	2888		Lipids and proteins (CH_2 asymmetric stretching)	(28)
2931	2931	2931	CH_2 asymmetric stretching	(28)
2936			Chain end CH_3 symmetric band	(28)
2960	2960	2960	Out-of-plane chain end antisymmetric CH_3 stretching	(28)
3010	3010	3010	Unsaturated =CH stretching	(28)

Supplementary Fig. S4. Mean Raman spectra ±1 standard deviations demonstrating the intra-variability for the case N1 (PTC follicular variant), N13 (adenoma), and N2 (healthy tissue).

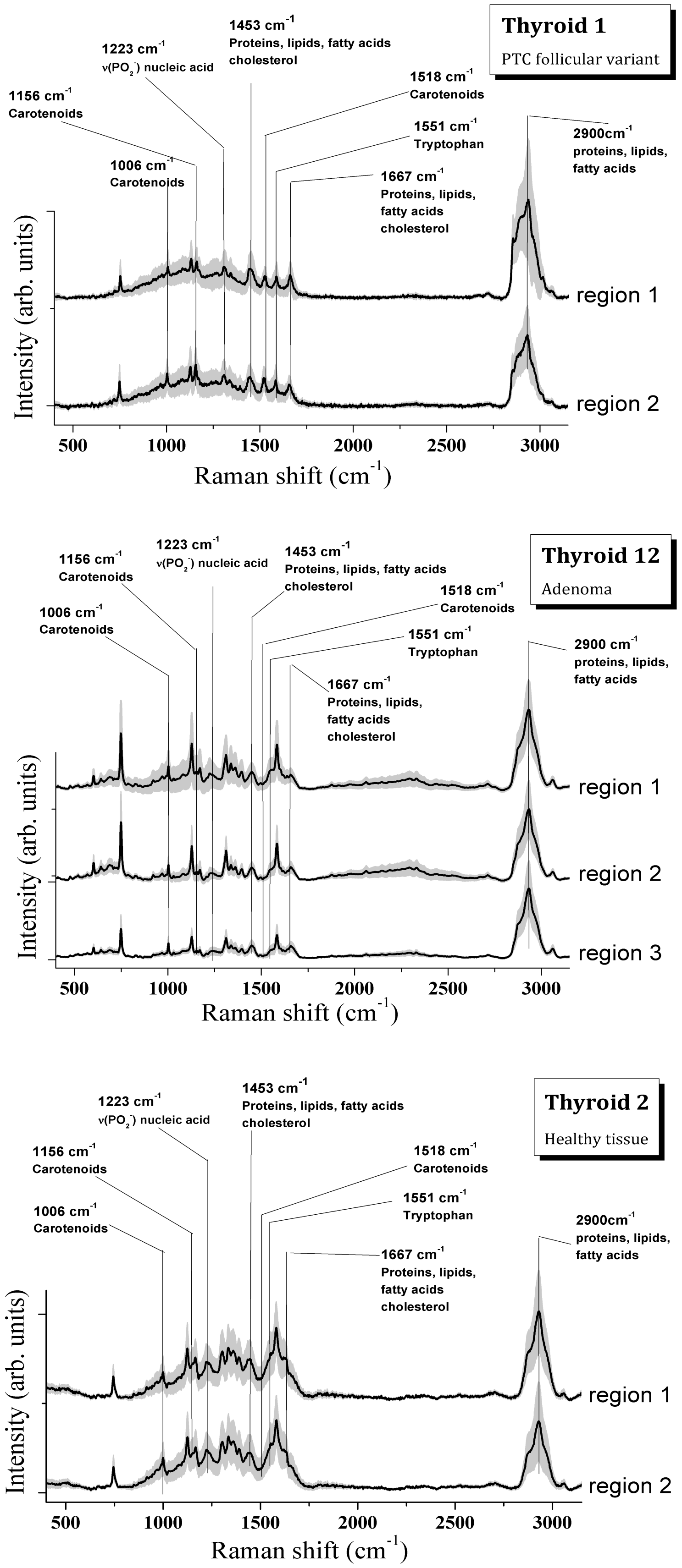

Supplement: Supplementary file 1 — Supplementary Information [file 41598_2017_14872_MOESM1_ESM.pdf]
